# Epoxyazadiradione suppresses breast tumor growth through mitochondrial depolarization and caspase-dependent apoptosis by targeting PI3K/Akt pathway

**DOI:** 10.1186/s12885-017-3876-2

**Published:** 2018-01-08

**Authors:** Dhiraj Kumar, Saikat Haldar, Mahadeo Gorain, Santosh Kumar, Fayaj A. Mulani, Amit S. Yadav, Lucio Miele, Hirekodathakallu V. Thulasiram, Gopal C. Kundu

**Affiliations:** 1 Laboratory of Tumor Biology, Angiogenesis and Nanomedicine Research, National Centre for Cell Science (NCCS), Pune, 411007 India; 2 Chemical Biology Unit, Division of Organic Chemistry, CSIR-National Chemical Laboratory, Pune, 411008 India; 3 Department of Biochemistry and Molecular and Cellular Biology, Georgetown University Medical Center, Washington D.C., 20057 USA; 4 Department of Genetics, LSU Health Sciences Center, New Orleans, LA 70112 USA

**Keywords:** Breast cancer, Apoptosis, PI3K, Metastasis, Angiogenesis, Limonoids

## Abstract

**Background:**

Breast cancer is one of the most commonly diagnosed invasive cancers among women around the world. Among several subtypes, triple negative breast cancer (TNBC) is highly aggressive and chemoresistant. Treatment of TNBC patients has been challenging due to heterogeneity and devoid of well-defined molecular targets. Thus, identification of novel effective and selective agents against TNBC is essential.

**Methods:**

We used epoxyazadiradione to assess the cell viability, mitochondrial potential, ROS level, cell migration, apoptosis and protein expression in cell culture models of TNBC MDA-MB-231 and ER+ MCF-7 breast cancer cells. The molecular mechanism was examined in two different type of breast cancer cells in response to epoxyazadiradione. We have also analyzed the effect of epoxyazadiradione on breast tumor growth using in vivo mice model.

**Results:**

In this study, we for the first time investigated that out of 10 major limonoids isolated from *Azadirachta indica*, epoxyazadiradione exhibits most potent anti-cancer activity in both TNBC and ER+ breast cancer cells. Epoxyazadiradione induces apoptosis and inhibits PI3K/Akt-mediated mitochondrial potential, cell viability, migration and angiogenesis. It also inhibits the expression of pro-angiogenic and pro-metastatic genes such as Cox2, OPN, VEGF and MMP-9 in these cells. Furthermore, epoxyazadiradione attenuates PI3K/Akt-mediated AP-1 activation. Our in vivo data revealed that epoxyazadiradione suppresses breast tumor growth and angiogenesis in orthotopic NOD/SCID mice model.

**Conclusion:**

Our findings demonstrate that epoxyazadiradione inhibits PI3K/Akt-dependent mitochondrial depolarisation, induces apoptosis and attenuates cell migration, angiogenesis and breast tumor growth suggesting that this compound may act as a potent therapeutic agent for the management of breast cancer.

**Electronic supplementary material:**

The online version of this article (10.1186/s12885-017-3876-2) contains supplementary material, which is available to authorized users.

## Background

Breast cancer is one of the most aggressive endocrine related cancer which has been considered as common malignancy affecting female worldwide. In spite of numerous therapeutic agents available to treat breast cancer, development of chemoresistance and recurrence of disease is frequently observed day by day [1]. Although several potent cytotoxic, hormonal and estrogen receptor (ER) targeted agents have been developed for treatment of breast cancer, the disease free survival of the patients remains unsatisfactory [2, 3]. Moreover, several breast cancer-targeted agents are available for effectively treating ER+ breast cancer [4, 5]. However, treatment of triple-negative breast cancer (TNBC) patients lack estrogen receptor (ER), progesteron receptor (PR) and human epidermal growth factor receptor 2 (HER2) has been challenging due to heterogeneity and devoid of well-defined molecular targets [6, 7]. About 20% of breast cancer patients are TNBC and commonly observed in younger patients [8]. Thus, identification of novel effective and selective agents against TNBC that do not produce considerable side effect is essential at this stage.

Neem (*Azadiracta indica*) plant is well-known for its diverse applications in traditional medicine in Indian subcontinent for many years. Various parts of this tree are being used over the years as the home-made remedies for several pathological conditions including hyperglycaemia, ulcer, malaria, cancer and dermatological complications [9, 10]. Structural diversity in the secondary metabolites of neem plant and more importantly their insecticidal efficacy and pharmacological activities has been explored in last five decades [11]. Over 150 triterpenoids have been isolated and structurally characterized from neem plant, majority of which belongs to tetranortriterpenoids (limonoids) [11]. On the basis of structural diversity, neem limonoids can be classified broadly into two groups; (i) basic/ring-intact limonoids possessing 4,4,8-trimethyl-17-furanylsteroidal skeleton (e.g. azadirone, azadiradione, gedunin etc.) and (ii) C-seco limonoids with rearranged framework generated through C-ring opening (e.g. salannin, nimbin, azadirachtin A etc) [11, 12]. Various neem limonoids including nimbolide, azadirachtin A, gedunin, azadirone and several other ring-intact limonoids have been tested for their cytotoxic potency against various cancer cell lines in vitro [13–17]. However, mode of action and anti-carcinogenic activity of these compounds under in vivo conditions are not well-explored. Our continuous effort to search for potent anti-carcinogenic plant-derived metabolites has prompted us to screen the neem limonoids against breast cancer cell lines and further investigate the molecular mechanism underlying this process. Previous studies have shown that neem-derived epoxyazadiradione limonoid exhibits anti-feedant properties [18]. Further, it has been shown that epoxyazadiradione acts as anti-inflammatory agent by attenuating macrophage migration inhibitory factor (MIF)-mediated macrophage migration [19]. Moreover, anti-cancer activity of epoxyazadiradione limonoids is not studied well. We report that epoxyazadiradione acts as anti-cancer agent in both TNBC and ER+ breast cancer models.

Several results revealed that mitochondria play crucial role in apoptosis through reactive oxygen species (ROS), apoptosis inducing factor (AIF) or caspase activation [20–23]. Phosphatidylinositol-3-kinase (PI3K)/Akt, MEK/ERK, GSK and STAT3, FAK and Src-mediated signaling play major role in breast cancer progression [24–27]. However, PI3K/Akt signaling pathway exhibits significant role in various aspect of tumor progression such as cell cycle progression, apoptosis, oncogenic transformation, cytokine production and activation of AP-1 and NF-κB [28]. Earlier report suggests that several components of PI3K/Akt pathway are dysregulated due to amplification, mutation and translocation more frequently in cancer patients [29]. This warrants the significant role of PI3K/Akt pathway in cancer specific drug development. Previous studies have shown that epoxyazadiradione inhibits the NF-κB activation and regulates pro-inflammatory cytokine production in RAW 264.7 cells [19]. Further, studies showed that blocking of PI3K/Akt and MEK signaling pathways are involved in induction of apoptosis and suppression of breast tumor growth [24, 25, 30].

In this context, we report the potential anti-cancer activities of neem-derived limonoid epoxyazadiradione under in vitro and in vivo conditions. It is noteworthy that out of ten major limonoids, epoxyazadiradione is highly potent cytotoxic agent. It induces apoptosis in both TNBC and ER+ breast cancer cells through mitochondrial-dependent caspase 3 and 9 activation. We have also shown that epoxyazadiradione induces apoptosis through ROS and AIF independent manner. Our findings suggest that it significantly attenuates breast cancer cell viability, migration and angiogenesis. It inhibits PI3K/Akt-mediated AP-1 activation and suppresses the expression of MMP-9, Cox2, OPN and VEGF leading to attenuation of breast tumor growth, angiogenesis and metastasis. Taken together, our study demonstrates that epoxyazadiradione may act as a potential therapeutic agent for control of TNBC and ER+ breast cancers.

## Methods

### Isolation and purification of neem limonoids

Ten major neem limonoid compounds (**1**: Epoxyazadiradione; **2**: Azadiradione; **3**: 17β-hydroxyazadiradione; **4**: Gedunin; **5**: Nimbin **6**: 6-Deacetylnimbin; **7**: Salannin; **8**: 3-Deacetylsalannin; **9**: Azadirachtin A; **10**: Azadirachtin B) were extracted and purified from *Azadirachta indica* as described earlier [12, 19]. Drugs were solubilized in DMSO and DMSO was used as vehicle control.

### Cell cultures and transfection

Human breast cancer cells, MDA-MB-231 and MCF-7 and normal human breast epithelial cells, MCF-10A were obtained from American Type Culture Collection (ATCC, Manassas, VA, USA). Cells were cultured as per standard conditions. pcDNA6-HA-Akt1 was transiently transfected in MDA-MB-231 cells using Dharmafect-1 (Dharmacon International) as per manufacturer’s instructions.

### MTT assay

To determine the cytotoxic effect of neem-derived limonoids, MTT assay was performed as described [24]. Briefly, MDA-MB-231 and MCF-7 (1 × 10^4^ cells/well) cells were plated in 96-well flat-bottom microplate. Further, cells were treated with all ten neem-derived limonoids independently at 100 μM and 200 μM for 24 h. MTT was added into each well and incubated at 37 °C for 4 h. After incubation, formazan crystals were dissolved with isopropanol and optical density of formazan solution, as a measure of cell viability was observed using a microplate reader at 570 nm (Thermo Scientific). In separate experiments, MDA-MB-231, MCF-7 and MCF-10A cells were independently treated with epoxyazadiradione (0–200 μM) in time-dependent manner and cytotoxic effect was determined by MTT assay as described above. In other experiments, MDA-MB-231 cells were pre-treated with Caspase 9 inhibitor-I (Calbiochem) or ROS scavenger agents, catalase (CAT) or N-acetyl-cysteine (NAC) (Sigma) independently for 1 h and further incubated with epoxyazadiradione (150 μM) for 24 h and MTT assay was performed.

### Annexin V/propidium iodide staining

MDA-MB-231 cells were treated with/without epoxyazadiradione (0–150 μM) for 24 h and stained with annexin V-FITC followed by propidium iodide (PI) and apoptosis was studied using apoptosis detection kit (BD Pharmingen) according to the manufacturer’s instructions. Stained cells were analyzed by FACSCalibur cytometer (BD Biosciences). In separate experiments, the effect of epoxyazadiradione on cell-cycle analysis was studied using PI staining as described [24]. Briefly, MCF-7 cells were treated with epoxyazadiradione (0–150 μM) for 24 h, stained with PI and analyzed on FACSCalibur cytometer. The cell cycle distribution was analyzed using CellQuest software (BD Immunocytometry System).

### Immunofluorescence study

Cells were grown on cover slips, treated in absence or presence of epoxyazadiradione with increasing concentrations (0–150 μM) for 24 h and immunofluorescence analysis was performed as described [31]. MDA-MB-231 or MCF-7 cells were fixed with 2% paraformaldehyde, blocked with 10% FBS and incubated with anti-c-Jun, anti-c-Fos or anti-AIF (Santa Cruz Biotechnology) antibody for overnight followed by fluorescence conjugated Cy2 or Cy3 (Calbiochem) specific antibody. To study the actin cytoskeleton reorganization, epoxyazadiradione treated MDA-MB-231 or MCF-7 cells were stained with FITC conjugated phalloidin (Sigma). Nuclei were stained with DAPI and analyzed under confocal microscope (Zeiss).

### TUNEL assay

To analyze the DNA fragmentation in response to epoxyazadiradione, TUNEL assay was conducted using APO-DIRECT™ Kit (BD Pharmingen) in MDA-MB-231 cells as per manufacturer’s instructions. Images were captured using fluorescence microscope (Leica).

### Determination of ROS production

To measure the effect of epoxyazadiradione on intracellular ROS production, MDA-MB-231 or MCF-7 cells were independently treated with increasing concentrations of epoxyazadiradione (0–150 μM) for 24 h. These cells were then stained with dihydroethidine (DHE) (Molecular Probes) for 20 min at 37 °C and analyzed on FACSCanto cytometer (BD Biosciences).

### Measurement of mitochondrial membrane potential (_∆_ψ_m_)

To examine the effect of epoxyazadiradione on mitochondrial membrane potential which is a crucial event in caspase-mediated apoptosis [32], MDA-MB-231 or MCF-7 cells were independently treated with epoxyazadiradione at different doses (0–150 μM) and stained with 5,5′,6,6′-tetrachloro-1,1′,3,3′-tetraethyl-benzamidazolocarbocyanin iodide (JC-1) (Molecular Probes) at 37 °C for 20 min and washed. The JC-1 aggregates, (healthy cells with functional mitochondria) and monomers, (apoptotic or unhealthy cells with collapsed mitochondria) were measured on FACSCanto cytometer (BD Biosciences). In separate experiments, MDA-MB-231 cells were treated with either perifosine or epoxyazadiradione and stained with JC-1. In another experiment, Akt1 overexpressed MDA-MB-231 cells were treated with epoxyazadiradione and stained with JC-1 and analyzed as described above.

### Immunoblot analysis

MDA-MB-231 or MCF-7 cells were treated with epoxyazadiradione (0–150 μM) for 24 h, lysed in lysis buffer and lysates containing equal amount of total proteins (40 μg) were resolved by SDS-PAGE and blotted onto nitrocellulose membranes as described [33]. The levels of apoptosis specific molecules such as Bax, Bad, Bcl2 (Santa Cruz Biotechnology), PARP, cleaved Caspase 9 and cleaved Caspase 3 (Cell Signaling Technology), metastasis and angiogenesis specific molecules such as Cox2, Flk1, VEGF (Santa Cruz Biotechnology) and cell signaling molecules such as OPN (Abcam), PI 3 kinase (p85 subunit) and p-Akt (Cell Signaling Technology), c-Jun and c-Fos (Santa Cruz Biotechnology) were analyzed using their specific antibodies. Actin was used as a loading control. All details of antibodies used are described in Additional file 1: Table S1.

### Wound and Transwell migration assays

To check the effect of epoxyazadiradione on breast cancer cell migration, wound and Transwell migration assays were performed as described [34]. Briefly, MDA-MB-231 cells were grown in monolayer and synchronized in serum free medium for 24 h and pretreated with caspase inhibitor (Sigma) for 1 h to avoid apoptosis induced by epoxyazadiradione and migration assay was performed. Wound with uniform size was made using sterile tip and the cells were treated with epoxyazadiradione at concentrations of 0–20 μM. Wound photographs were captured at *T* = 0 and *T* = 12 h using phase contrast microscope (Nikon), distance migrated was measured and analyzed (Image-Pro plus software) and represented as bar graph (Sigma Plot 10.0 software). In another experiments, to examine the involvement of PI3K/Akt on cell migration, MDA-MB-231 cells were either treated with perifosine (Akt inhibitor) [35] or epoxyazadiradione or transfected with pcDNA6-HA-Akt1 and then treated with epoxyazadiradione and wound assay was performed as describe above.

In separate experiments, cell migration assay was performed using Transwell Boyden chamber (Corning) at above conditions as described earlier [36]. Briefly, MDA-MB-231 cells (1 × 10^5^) were pretreated with epoxyazadiradione or perifosine or transfected with pcDNA6-HA-Akt1 followed by treatment with epoxyazadiradione and used in the upper portion of Boyden chamber. In the lower chamber, 5% FBS was used as chemoattractant. Cells were incubated further at 37 °C for 12 h, the migrated cells to the lower surface of the Transwell membrane were fixed with 4% paraformaldehyde for 10 min and stained with 5% Crystal Violet in 25% methanol for 10 min and washed. Migrated cells were photographed at five high power fields (hpf) under inverted microscope at magnifications of 10X (Nikon), counted, analyzed statistically and represented graphically (Sigma Plot 10.0 software).

### In vitro tube formation assay

To examine the effect of epoxyazadiradione on angiogenesis, tube formation assay was performed with HUVECs as described [34]. Briefly, HUVECs (Lonza) were seeded (1 × 10^4^) onto Matrigel pre-coated 96-well plate and treated with epoxyazadiradione (0–20 μM) and used for tube formation assay. After 8 h, tube like structures were observed and photographed using a phase contrast microscope (Nikon).

### Zymography

To examine the effect of epoxyazadiradione on MMP-9 activity, gelatinolytic assay was performed as described previously [37]. Briefly, MDA-MB-231 cells were treated with epoxyazadiradione (0–150 μM) for 24 h in basal medium. Conditioned medium (CM) was collected, dialyzed, lyophilized and CM containing equal amount of total proteins was loaded on gelatin gel and gelatinolytic activity of MMP-9 was studied.

### Electrophoretic mobility shift assay (EMSA)

To determine the role of epoxyazadiradione on AP-1-DNA binding, EMSA was performed as described earlier [38, 39]. Briefly, MDA-MB-231 cells were treated with different concentration of epoxyazadiradione (0–150 μM). After 24 h, cells were washed and nuclear extracts were prepared and incubated with γ-^32^P-labeled double-stranded oligonucleotide containing AP-1 consensus sequence (5’-CGC TTG ATG ACT CAG CCG GAA-3′) in binding buffer (100 mM Tris-HCl, 500 mM NaCl, 10 mM DTT, 50% Glycerol) with 1 mg/ml BSA and 1 μg sonicated salmon sperm DNA for 30 min at room temperature. AP-1-DNA complex were resolved on native gel electrophoresis (8%). The gel was dried and exposed to an X-ray film overnight at −80 °C for autoradiography.

### Tumor xenograft and IVIS analysis

All mice experiments were performed according to the institutional guidelines, following a protocol approved by the Institutional Animal Care and Use Committee (IACUC) of National Centre for Cell Science (NCCS), Pune, India. MDA-MB-231-Luc (2 × 10^6^) cells were mixed with Matrigel (1:1) (BD Biosciences) and administered orthotopically into mammary fat pad of 6-week old female non-obese diabetic/severe combined immunodeficient (NOD/SCID) mice. Once tumor formed, mice were randomly divided into three groups. Further, two doses of epoxyazadiradione (25 mg/Kg and 100 mg/Kg body wt) was injected intraperitoneally (i.p.) twice a week into these mice. Tumors length and breadth were measured twice a week using Vernier Calipers. Tumor volumes were calculated by using the formula, V = π/6 [(l x b)^3/2^]. In vivo bioluminescence imaging was conducted using Living Image acquisition and analysis software on a cryogenically cooled In Vivo Imaging System (IVIS) (Xenogen Corp.) as described earlier [40]. At the end of experiments, mice were sacrificed and tumor samples were removed, photographed, weighed and fixed in formalin. Further, these tumor sections were stained with H & E and analyzed by immunohistochemistry using anti-VEGF antibody.

### Statistical analysis

The data were expressed as mean ± SEM using Sigma Plot 10.0 software. The levels of significance were calculated using unpaired Student’s t test or a one-way ANOVA test. A ‘p’ value less than 0.05 (*p* < 0.05) was considered as statistically significant.

## Results

### Neem-derived limonoids differentially inhibit the breast cancer cell viability

**Fig. 1:**
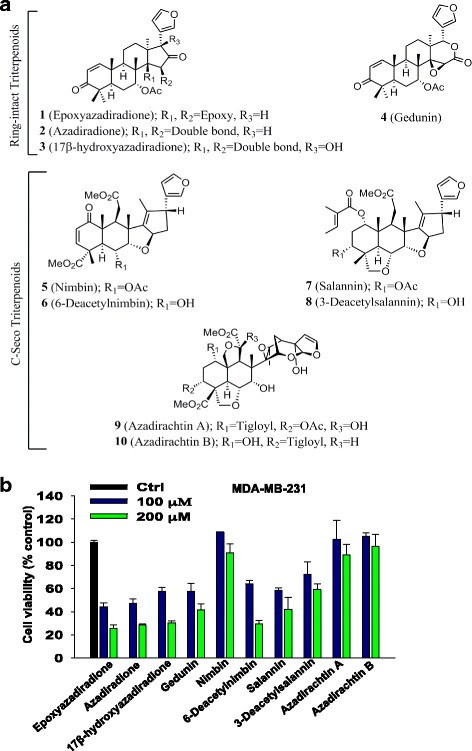
Epoxyazadiradione a neem limonoid exhibits potent cytotoxic effect in breast cancer cells: **a** Structure of ten major limonoids isolated from neem. **b** MDA-MB-231 cells were seeded into 96-well plate at density of 1 × 10^4^ cells and treated with ten major neem limonoids (1: Epoxyazadiradione; 2: Azadiradione; 3: 17β-hydroxyazadiradione; 4: Gedunin; 5: Nimbin; 6: 6-Deacetylnimbin; 7: Salannin; 8: 3-Deacetylsalannin; 9: Azadirachtin A; 10: Azadirachtin B) for 24 h at 100 and 200 μM and cell death was analyzed using MTT assay. The inhibition of the percentage of cell viability is represented into bar graph. Values are represented in mean ± SEM of three independent experiments

Several anti-cancer therapies are available for the treatment of breast cancer. However, they are relatively ineffective against triple negative breast cancer (TNBC). To target these TNBC specific cells, we have extracted and purified 10 major limonoids including **1**: Epoxyazadiradione; **2**: Azadiradione; **3**: 17β-hydroxyazadiradione; **4**: Gedunin; **5**: Nimbin; **6**: 6-Deacetylnimbin; **7**: Salannin; **8**: 3-Deacetylsalannin; **9**: Azadirachtin A; **10**: Azadirachtin B from neem plant (Fig. 1a). To examine their anti-cancer effect, these major neem limonoids (**1** to **10**) are tested in MDA-MB-231 and MCF-7 breast cancer cells using MTT assay. Our data showed the wide variation in their potency (Fig. 1b and Additional file 1: Figure S1a). It was observed that limonoids with ring-intact (basic) skeletons exibit high cytotoxic effect compared with C-Seco. The investigated basic limonoids share common structural characteristics in A, B and C rings. Their structural diversity appears from the variation in D-ring. Epoxyazadiradione (**1**) contains five-member D-ring ketone (C-16) with 14,15β-epoxide and showed highest cytotoxicity among all the limonoids when tested in MDA-MB-231 and MCF-7 cells (Fig. 1b and Additional file 1: Figure S1a). Reduction of the 14,15β-epoxide as observed in azadiradione (**2**) and 17β-hydroxyazadiraione (**3**) resulted in lowering of cytotoxicity. Also, lactonization of the D-ring (Gedunin, **4**) in epoxyazadiradione structure reduced the cytotoxic effect in these cell lines. The C-seco limonoids of salannin and nimbin skeleton showed diminished activity as compared to ring-intact limonoids. Further, rearranged and highly complex skeletons of azadirachtins (**9** and **10**) showed less cytotoxic activity in these cell lines (Fig. 1b and Additional file 1: Figure S1a). Among all the limonoids tested for their cytotoxic effects, epoxyazadiradione (**1**) was selected further for its molecular mechanism and anti-cancer effect using in vitro and in vivo models.

### Epoxyazadiradione attenuates breast cancer cell viability and induces apoptosis

**Fig. 2:**
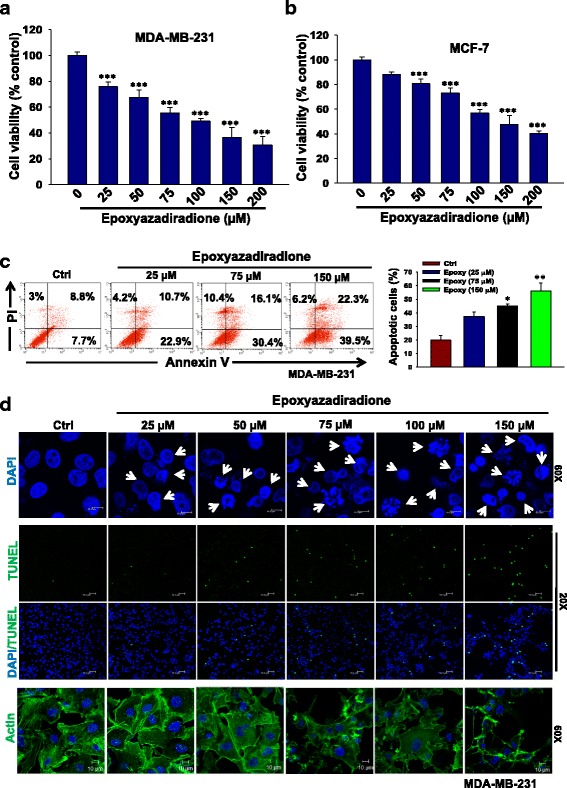
Epoxyazadiradione inhibits cell viability in TNBC and ER+ breast cancer cells through induction of apoptosis: **a** and **b** MDA-MB-231 (1 × 10^4^) and MCF-7 (1 × 10^4^) cells were seeded into 96-well plate and treated with increasing concentrations of epoxyazadiradione (0–200 μM) for 24 h. Cell death induced by epoxyazadiradione was analyzed by MTT assay. The percentage inhibition of cell viability represented graphically. **c** MDA-MB-231 cells were treated with epoxyazadiradione at indicated doses for 24 h and cells were stained with annexin V-FITC and propidium iodide and analyzed by flow cytometer. The percentage of apoptotic cells were quantified and represented into bar graph from three independent experiments. **d** Chromatin condensation or nuclear fragmentations were analysed by DAPI staining or TUNEL assay using fluorescence microscope in MDA-MB-231 cells in presence of epoxyazadiradione at indicated concentrations. Arrows indicate the chromatin condensation and nuclear fragmentation. The actin disorganization was examined in epoxyazadiradione treated MDA-MB-231 cells using phalloidin FITC staining and analyzed by confocal microscopy. Values are represented in mean ± SEM of three independent experiments in each case or representative of a typical experiment: *, *p* < 0.02; **, *p* < 0.006; ***, *p* < 0.0004 by one-way ANOVA

Our previous results demonstrate that epoxyazadiradione is highly toxic against MDA-MB-231 and MCF-7 cells. To further confirm the cytotoxic effect of epoxyazadiradione in a concentration and time-dependent manner, cells were treated with epoxyazadiradione (0–200 μM) for 24, 48, 72 and 96 h and cell viability was determined by MTT assay. The results revealed that epoxyazadiradione significantly inhibits cell viability of MDA-MB-231 and MCF-7 cells in a dose- and time-dependent manner (Fig. 2a and b, Additional file 1: Figure S1b, S1c and Table S2). We have also analyzed the cytotoxic effect of epoxyazadiradione on MCF-10A cells and found that this compound has very less cytotoxic effect as compared to MDA-MB-231 and MCF-7 cells (Fig. 2a and b and Additional file 1: Figure S1d). Further, we performed cell cycle analysis in MCF-7 and the data revealed that percentage of cells in G2/M phases is increased significantly in response to epoxyazadiradione (Additional file 1: Figure S1e). These data revealed that reduction in breast cancer cell viability by epoxyazadiradione may be associated with cell cycle arrest at G2/M phase.

To determine whether reduction in cell viability is associated with apoptosis, we treated MDA-MB-231 cells with increasing doses of epoxyazadiradione (0–150 μM) for 24 h, stained with annexin V-FITC and PI and analyzed by flow cytometry. The data revealed that epoxyazadiradione induces apoptosis significantly (Fig. 2c). In order to examine the effect of epoxyazadiradione on chromatin condensation and nuclear fragmentations, TUNEL assay was performed. In separate experiments, to examine the effect of this compound on destruction of cell integrity through actin disorganization, MDA-MB-231 and MCF-7 cells were stained with phalloidin FITC. The results showed a typical apoptotic nuclei, chromatin condensation or cell blebbing and loss of cell integrity were observed in these cells (Fig. 2d and Additional file 1: Figure S2a, S2b).

### Epoxyazadiradione induces apoptosis through mitochondrial dysfunction in breast cancer cells

**Fig. 3:**
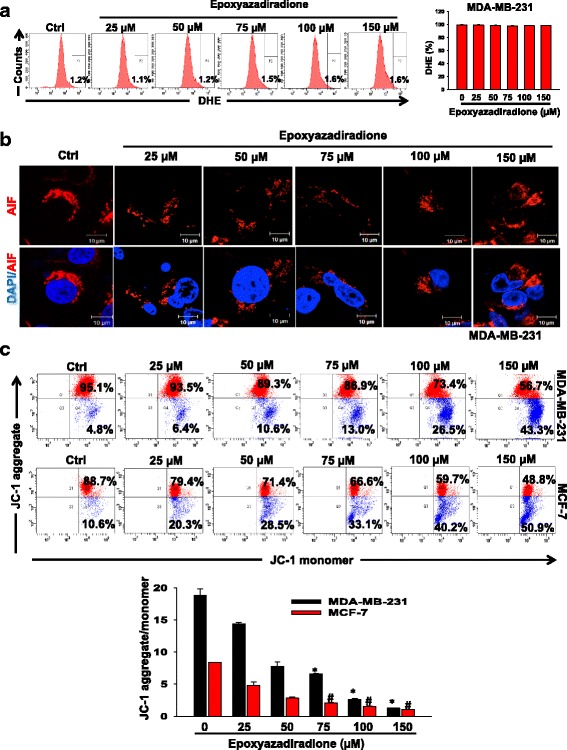
Epoxyazadiradione induces apoptosis through disruption of mitochondrial homeostasis: **a** MDA-MB-231 cells were treated with increasing concentration of epoxyazadiradione for 24 h. Cells were stained with DHE at 37 °C for 20 min and fluorescence intensity was analyzed by flow cytometry and represented as histogram. The percentage of DHE staining as compared to unstained cells was quantified and represented graphically. **b** MDA-MB-231 cells were treated with epoxyazadiradione for 24 h at indicated concentrations, stained with anti-AIF antibodies and analyzed by confocal microscopy. Nuclei were stained with DAPI. **c** MDA-MB-231 (upper panel) and MCF-7 (lower panel) cells were independently treated with epoxyazadiradione with different doses (0–150 μM) and JC-1 staining was performed at 37 °C for 20 min. JC-1 aggregates (red fluorescence) and JC-1 monomers (green fluorescence) in stained cells were analyzed by flow cytometry. The ratio of JC-1 aggregates and JC-1 monomers was analyzed statistically and represented graphically from three independent experiments. Values are represented in mean ± SEM. *, *p* < 0.007; #, *p* < 0.0008 by Student’s *t* test

Apoptosis is mediated by several pathways such as ROS-dependent, apoptosis inducing factor (AIF) and caspase-mediated pathway. To examine the effect of epoxyazadiradione on translocation of AIF into nucleus, mitochondrial membrane potential and ROS generation, MDA-MB-231 and MCF-7 cells were treated with this compound and independently stained with AIF, DHE and JC-1 and analyzed by immunofluorescence or flow cytometry. The data showed that epoxyazadiradione does not affect the ROS level or translocation of AIF into the nucleus in these cells as compared with control (Fig. 3a, b and Additional file 1: Figure S2a, S2c). These data confirmed that epoxyazadiradione-induced apoptosis is independent of ROS or AIF.

The loss of mitochondrial membrane potential is another hallmark of apoptosis. Accordingly, MDA-MB-231 and MCF-7 cells were treated with epoxyazadiradione and then stained with JC-1 dye. The aggregated (red fluorescent) and monomer (green fluorescent) forms of JC-1 were analyzed by flow cytometry. The results demonstrated that this compound decreased the intensity of red and increased the intensity of green fluorescence in dose-dependent manner, as would be expected for apoptotic cell death through reduction of mitochondrial membrane potential (Fig. 3c). Taken together, these data demonstrate that epoxyazadiradione induces apoptosis through mitochondrial dysfunction but not through ROS or AIF-mediated pathway.

### Caspase 3 and 9 are involved in epoxyazadiradione-induced apoptosis

**Fig. 4:**
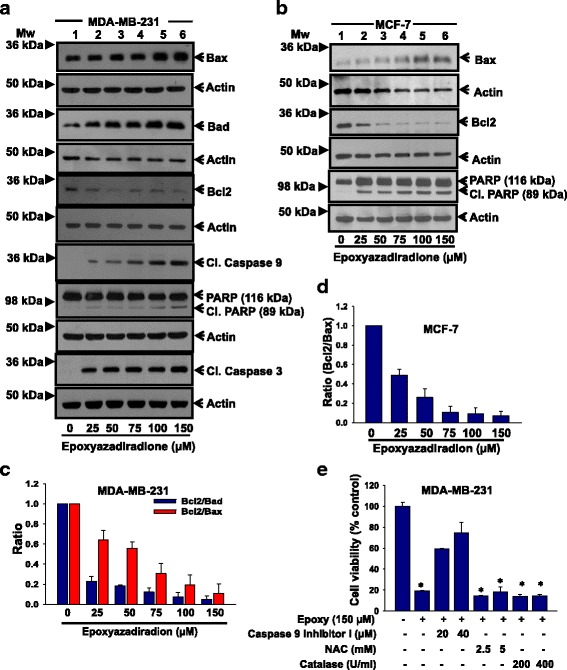
Epoxyazadiradione augments apoptosis in breast cancer cells through Caspase-dependent and ROS independent pathway: **a** MDA-MB-231 cells were treated with epoxyazadiradione (0–150 μM) for 24 h and the expression of Bcl2 family proteins including Bax, Bad and Bcl2 and cleaved caspase 9, 3 and PARP were analyzed by immunoblot. Actin was reprobed with each respective original blot and used as loading control. **b** The expression of Bcl2 family protein including Bax and Bcl2 and PARP were analyzed by western blot in MCF-7 cells upon treatment with epoxyazadiradione at indicated doses. **c** The ratios of Bcl2 with Bad or Bax were represented by densitometric quantification of western blot from three independent experiments in MDA-MB-231 cells. **d** The ratio of Bcl2 and Bax was represented by densitometric quantification of western blot from three independent experiments and represented graphically in MCF-7 cells. **e** MDA-MB-231 cells were either independently pretreated with caspase 9 inhibitor I (20 and 40 μM) or N-acetyl cysteine (NAC, 2.5 and 5 mM) or catalase (200 and 400 U/ml) for 1 h followed by treatment with epoxyazadiradione (150 μM) for another 24 h. The cell viability was analyzed using MTT assay. Values are represented in mean ± SEM of three independent experiments; *, *p* < 0.00004

The Bcl2 family members are known to play vital role in regulation of cytochrome C release [41]. Therefore, we further evaluated the mechanism by which epoxyazadiradione mediates apoptosis. Accordingly, we examined the expression of anti-apoptotic (Bcl2) and pro-apoptotic (Bad and Bax) specific proteins by western blot. The data showed that epoxyazadiradione attenuates Bcl2 and augments Bad and Bax expressions in MDA-MB-231 and MCF-7 cells in a dose-dependent manner (Fig. 4a, b). The results revealed that the ratio of Bcl2/Bad or Bcl2/Bax was decreased upon treatment with epoxyazadiradione in MDA-MB-231 and MCF-7 cells (Fig. 4c, d). Apaf-1 binds with Caspase 9 in presence of cytochrome C that leads to Caspase 9 activation. Activated Caspase 9 then cleaves and further activates Caspase 3 [42]. Accordingly, we have examined the levels of cleaved Caspase 3 and 9 in response to epoxyazadiradione by western blot. The data revealed that epoxyazadiradione induces cleaved Caspase 9 and 3 in a dose-dependent manner (Fig. 4a). This compound also induces PARP cleavage in MDA-MB-231 and MCF-7 cells (Fig. 4a, b).

To further examine whether epoxyazadiradione-induced apoptosis is caspase or ROS-mediated, MDA-MB-231 cells were pretreated with caspase 9 inhibitor I or ROS scavenger N-acetyl-cysteine (NAC) or catalase (CAT) and further incubated with epoxyazadiradione and MTT assay was performed. The data suggest that caspase 9 inhibitor restored the epoxyazadiradione-mediated cell viability significantly whereas NAC or CAT had no effect (Fig. 4e). Overall, these data emphasized that epoxyazadiradione induces apoptosis through mitochondria-mediated caspase 9 and 3 activation but not through ROS-dependent manner in breast cancer cells.

### Epoxyazadiradione inhibits breast cancer cell migration and endothelial tube formation

**Fig. 5:**
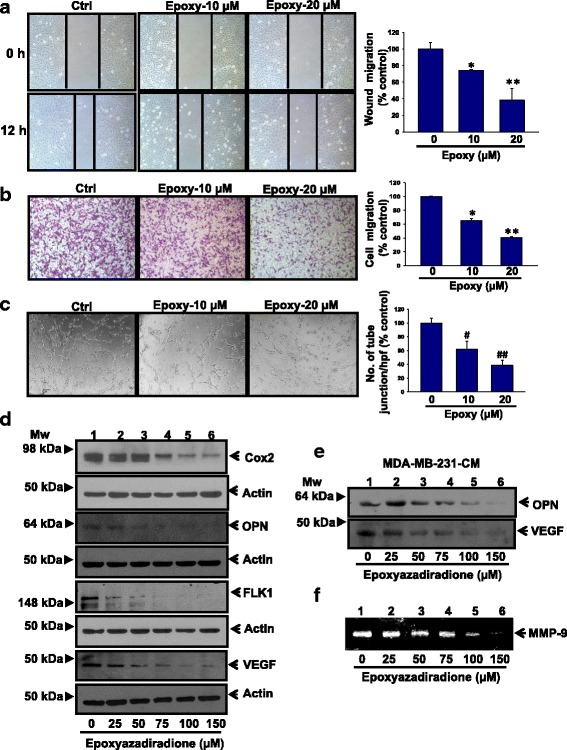
Epoxyazadiradione inhibits cell migration, HUVECs tube formation and pro-angiogenic and metastasis specific gene expression: **a** Confluent monolayer of MDA-MB-231 cells were pretreated with caspase inhibitor (40 μM) for 1 h, wounded with constant width and treated with epoxyazadiradione (0–20 μM) for another 12 h. Photographs of wound were taken at *T* = 0 and 12 h. Migrated distance were measured using Image-Pro plus and analyzed statistically and represented graphically using Sigma Plot. **b** MDA-MB-231 cells were pretreated with Caspase inhibitor (40 μM) for 1 h followed by treatment with epoxyazadiradione (0–20 μM) for another 12 h and added to the upper portion of Transwell chamber. After 12 h, migrated cells were stained with 5% crystal violet and photographed at five different high power field (hpf), counted and analyzed statistically and represented graphically. **c** Human umbilical vein endothelial cells (HUVECs) were treated with epoxyazadiradione at indicated doses and after 8 h, images of tubular structure were captured under phase contrast microscope, the number of tube junction/hpf were analyzed statistically and represented graphically. **d** The expressions of pro-angiogenic and metastasis specific molecules such as Cox2, OPN, Flk1 and VEGF were analyzed by western blot in MDA-MB-231 cells treated with epoxyazadiradione (0–150 μM) for 24 h. **e** Breast cancer cells (MDA-MB-231) were treated with epoxyazadiradione (0–150 μM) for 24 h and conditioned media (CM) were collected and expressions of OPN and VEGF were analyzed by western blot. **f** Conditioned media obtained from epoxyazadiradione (0–150 μM) treated MDA-MB-231 cells were analyzed for MMP-9 activity by zymography.Values are represented in mean ± SEM of three independent experiments or representative of a typical experiment: *, *p* < 0.008; **, *p* < 0.004; ^#^, *p* < 0.03; ^##^, *p* < 0.003 by Student’s *t* test with untreated control cells

To examine the effect of epoxyazadiradione on breast cancer cell migration, MDA-MB-231 cells were pretreated with caspase inhibitor followed by treatment with two doses of epoxyazadiradione. The results revealed that this compound attenuates cell migration as shown by wound scratch and Boyden chamber assays in a dose-dependent manner (Fig. 5a, b). These data are further quantified (Fig. 5a and b). In order to examine whether the change in migration in response to epoxyazadiradione is not due to proliferation, MDA-MB-231 cells were pretreated with mitomycin C and wound assay was performed in absence or presence of epoxyazadiradione. The results showed that epoxyazadiradione inhibits the migration irrespective of mitomycin C suggesting that inhibition of migration by this compound is not because of proliferation (Additional file 1: Figure S2d).

Several studies revealed that angiogenesis plays an important role in the maintenance of the aggressive nature of tumors [43]. Therefore, to determine whether epoxyazadiradione has any anti-angiogenic property in human umbilical vein endothelial cells (HUVECs), tube formation assay was performed. Our data demonstrate that epoxyazadiradione attenuates tubular like structure formation in HUVECs (Fig. 5c). `.

### Epoxyazadiradione attenuates the expression of angiogenesis and metastasis specific genes

Previous reports suggest that OPN regulates tumor progression and angiogenesis through regulation of VEGF, Cox2 and MMP-9 expression and activation in melanoma and breast cancer cells [37, 44, 45]. Therefore, we next examined the effect of epoxyazadiradione on endogenous expression of OPN, VEGF, Flk1 and Cox2 in MDA-MB-231 cells by western blot. The data revealed that Cox2, OPN, VEGF and Flk1 were downregulated in response to this compound in a dose-dependent manner (Fig. 5d). These observations were further validated in conditioned medium (CM) obtained from epoxyazadiradione treated MDA-MB-231 cells. Results showed that epoxyazadiradione attenuates the expression of secretary OPN and VEGF levels (Fig. 5e). MMP-9 is a pro-metastatic enzyme that is involved in degradation of extracellular matrix proteins (ECM) and controls metastasis [46, 47]. Accordingly, we have examined the level of MMP-9 activity in CM obtained from epoxyazadiradione treated MDA-MB-231 cells by zymography. The data depicts that this compound reduced the MMP-9 activity in a dose-dependent manner (Fig. 5f).

### Epoxyazadiradione downregulates PI3K/Akt and AP-1 activation in breast cancer cells

**Fig. 6:**
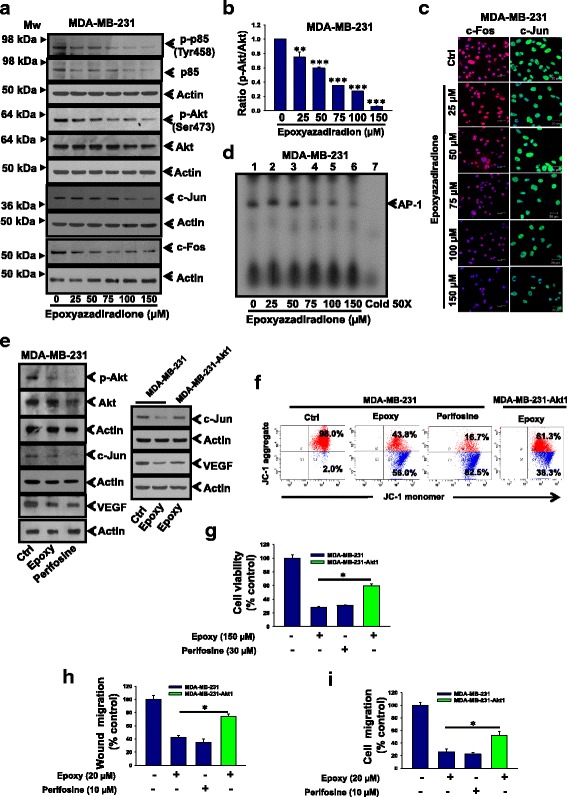
Epoxyazadiradione attenuates PI3K/Akt pathway-dependent AP-1 DNA binding, VEGF expression and breast cancer cell migration and induces apoptosis: **a** MDA-MB-231 cells were treated with epoxyazadiradione. The level of phosphorylation of p85 (Tyr458) and Akt (Ser473) and c-Jun and c-Fos were analyzed by immunoblot. Actin was independently reprobed with respective original blots and served as loading control. **b** Ratio of densitometric analysis of p-Akt and Akt. Values are represented in mean ± SEM of three independent experiments; **, *p* < 0.004; ***, *p* < 0.0004 with untreated control cells. **c** The expressions of c-Fos and c-Jun were detected by confocal microscopy at indicated conditions. **d** Nuclear fractions isolated from MDA-MB-231 cells treated with epoxyazadiradione for 24 h and used for EMSA. Nuclear extracts were incubated with γ-^32^P-labeled oligonucleotides containing the AP-1 consensus binding site. Arrow indicates the AP-1-DNA bound complex. **e** The expression of p-Akt, c-Jun and VEGF were analyzed by immunoblot in MDA-MB-231 cells treated with either epoxyazadiradione (50 μM) or perifosine (15 μM) for 24 h (left panel). MDA-MB-231 cells were transiently transfected with pcDNA6-HA-Akt1 and then treated with epoxyazadiradione (50 μM) for 24 h. The expression of c-Jun and VEGF was analyzed by western blot (right panel). **f** Flow cytometry analysis of JC-1 staining in MDA-MB-231 cells either treated with epoxyazadiradione (150 μM) or perifosine (30 μM) or overexpressed with Akt1 and then treated with epoxyazadiradione (150 μM) for 24 h. **g** MDA-MB-231 cells were either treated with epoxyazadiradione (150 μM) or perifosine (30 μM) or overexpressed with Akt1 and then treated with epoxyazadiradione (150 μM) for 24 h and cell viability was determined using MTT assay. Data were analyzed statistically and represented graphically. **h** Wound migration assay at indicated conditions. **i** Transwell migration assay as indicated conditions and cells. Values are represented in mean ± SEM of three independent experiments in each case or representative of a typical experiment. * *P* < 0.0005

We have further explored the mechanism by which epoxyazadiradione regulates cell migration, angiogenesis and apoptosis in MDA-MB-231 cells. PI3K/Akt pathway is highly involved in regulation of cell migration, apoptosis, tumor growth, EMT and metastasis in many aggressive cancers [30, 48, 49]. Kumar et al. have reported that Andrographolide inhibits cell migration through downregulation of PI3K/Akt signaling in MDA-MB-231 cells [24]. Therefore, we sought to determine whether epoxyazadiradione regulates PI3K/Akt pathway in MDA-MB-231 and MCF-7 cells. Our western blot data revealed that epoxyazadiradione downregulates phosphorylation of p85 and Akt drastically in a dose-dependent manner in these cells (Fig. 6a, b and Additional file 1: Figure S3a). We have then evaluated the expression of c-Jun and c-Fos in epoxyazadiradione treated cells by western blot and immunofluorescence. The results revealed that the expression of c-Jun and c-Fos was abrogated by epoxiazadiradione in these cells (Fig. 6a, c and Additional file 1: Figure S3a). Further, we have examined the role of epoxyazadiradione on AP-1-DNA binding by EMSA. Our data revealed that it suppresses AP-1-DNA binding in these cells (Fig. 6d). Overall, these results demonstrate that epoxyazadiradione downregulates PI3K/Akt and AP-1 activation in breast cancer cells.

### PI3K/Akt signaling is involved in epoxyazadiradione-induced mitochondrial dysfunction, apoptosis and migration inhibition in breast cancer cells

To further confirm whether Akt is involved in epoxyazadiradione-induced apoptosis, MDA-MB-231 cells were transiently transfected with pcDNA6-HA-Akt1 and then treated with epoxyazadiradione. In separate experiments, MDA-MB-231 cells were treated with perifosine or epoxyazadiradione. The expressions of p-Akt, c-Jun and VEGF were analyzed by immunoblot. The results showed that epoxyazadiradione or perifosine independently inhibits the expression of these molecules whereas cells overexpressed with Akt1 restored these effects (Fig. 6e and Additional file 1: Figure S3b). To further study the role of PI3K/Akt in regulation of mitochondrial homeostasis, MDA-MB-231 cells were overexpressed with Akt1 as described above. MDA-MB-231 cells alone or cells overexpressed with Akt1 were independently pretreated with epoxyazadiradione and then stained with JC-1. In separate experiments, MDA-MB-231 cells were pretreated with perifosine and then stained with JC-1. The data revealed that epoxyazadiradione or perifosine reduced the intensity of red fluorescence (JC-1 aggregates) significantly whereas overexpression of Akt1 enhanced the intensity of red fluorescence in response to epoxyazadiradione suggesting that this compound induces apoptosis through Akt-mediated mitochondrial dysfunction (Fig. 6f). Furthermore, our results suggest that perifosine or epoxyazadiradione independently inhibits cell viability and migration in MDA-MB-231 cells whereas cells overexpressed with Akt1 enhanced these phenomena in response to epoxyazadiradione (Fig. 6g-i and Additional file 1: Figure S3c). Overall, these data suggest that PI3K/Akt is involved in epoxyazadiradione-induced mitochondrial dysfunction, apoptosis and cell migration and regulates c-Jun and VEGF expression in MDA-MB-231 cells.

### Epoxyazadiradione suppresses breast tumor growth and angiogenesis using in vivo model

**Fig. 7:**
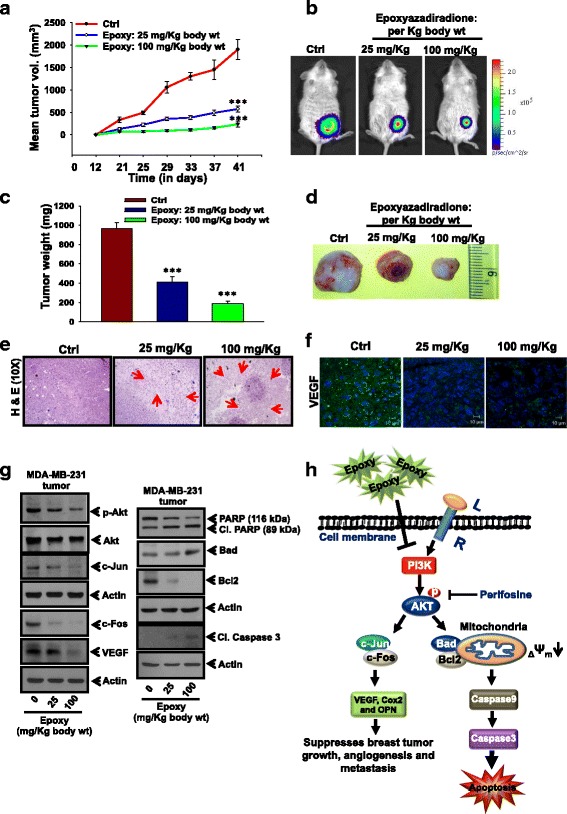
Epoxyazadiradione suppresses breast cancer growth and angiogenesis under in vivo conditions: **a** MDA-MB-231-Luc (2 × 10^6^) cells were injected orthotopically into NOD/SCID mice and then 25 and 100 mg/Kg body wt of epoxyazadiradione was injected intraperitoneally (i.p.) twice a week for 6 weeks. Tumor volumes were measured twice a week using Vernier Calipers, analyzed statistically and represented graphically (mean ± SEM, *n* = 5; ***, *p* < 0.0008 compared to untreated control tumor). **b** Photographs of bioluminescence imaging of representative tumor bearing NOD/SCID mice were analyzed using IVIS as indicated conditions. **c** and **d** Tumors were excised, photographed, weighed and analyzed statistically. Bar graph represents mean tumors weight (Mean ± SD, *n* = 5; ***, *p* < 0.0008). **e** Tumor sections derived from epoxyazadiradione treated or untreated mice were stained with H & E. Arrows indicate the necrotic area. **f** Tumor sections derived from epoxyazadiradione treated mice were analyzed for VEGF expression by confocal microscopy using anti-VEGF antibody. **g** Western blot analysis of p-Akt, c-Fos, c-Jun, VEGF and apoptosis associated molecules in the epoxyazadiradion treated tumor lysates. **h** Schematic representation of epoxyazadiradione regulated PI3K/Akt-mediated mitochondrial homeostasis, caspase-dependent apoptosis and attenuation of AP-1 activation, VEGF and MMP-9 expression and suppression of tumor growth and angiogenesis in breast cancer model

To investigate the effect of epoxyazadiradione on in vivo breast tumor growth in orthotopic mice model, MDA-MB-231-Luc cells were injected into mammary fat pad of NOD/SCID mice. After 10 days, tumor-bearing mice were randomly divided into three groups (5 mice each). Vehicle or two doses of epoxyazadiradione (25 mg/Kg body wt and 100 mg/Kg body wt) were injected intraperitoneally (i.p.) twice a week till 6 weeks. Tumor volumes were measured using Vernier Calipers twice a week (Fig. 7a). Further, tumor growth was monitored in a real-time manner using In Vivo Imaging System (IVIS) (Fig. 7b). At the end of experiments, mice were sacrificed; tumors were dissected, photographed and weighed (Fig. 7c, d). Interestingly, our data showed that epoxyazadiradione significantly reduced tumor volume and weight as compared with vehicle treated mice (Fig. 7a-d). Taken together, our in vivo data demonstrate that epoxyazadiradione attenuates breast tumor growth.

To further correlate our in vitro data with in vivo findings, tumor samples were sectioned and analyzed by histopathology and immunohistochemistry. The H & E staining of epoxyazadiradione treated tumor sections showed less infiltration of tumor cells and higher necrotic area (Fig. 7e). Next, we have examined whether reduction of tumor volume is associated with attenuation of angiogenesis and PI3K/Akt pathway-dependent c-Fos and c-Jun expression and induction of apoptosis. Accordingly, we have analyzed the expression of p-Akt, c-Jun, c-Fos, VEGF and apoptosis related molecules in epoxyazadiradione treated mice tumor tissues. Our results indicated that epoxyazadiradione suppresses VEGF expression compared with control as shown by immunohistochemistry and western blot (Fig. 7f, g). Further, epoxyazadiradione also attenuates the activation and expression of p-Akt, c-Jun and c-Fos (Fig. 7g). It induces the apoptosis in these tumors as indicated by elevated level of cleaved PARP, cleaved Caspase 3, Bad and downregulation of Bcl2 (Fig. 7g). Overall, these data demonstrate that epoxyazadiradione attenuates tumor growth through inhibition of PI3K/Akt-dependent c-Fos, c-Jun and VEGF expression and induction of apoptosis (Fig. 7h).

## Discussion

Despite of several drugs available for the treatment of breast cancer, emerging drug resistance leads to high mortality is observed in many cases. Hence, identification of novel and selective anti-cancer agents which exhibit potent anti-cancer activity and less side effects is essential for the treatment of TNBC and ER+ breast cancer.

In this study, we have screened the anti-cancer properties of 10 major neem-derived limonoids and found that epoxyazadiradione exhibits most potent anti-cancer activity. It induces cell death in both TNBC and ER+ breast cancer cells through attenuation of PI3K/Akt-mediated mitochondrial depolarization and induction of caspase-dependent apoptosis. Further, attenuation of PI3K/Akt pathway by epoxyazadiradione leads to inhibition of c-Jun and c-Fos expression and AP-1-DNA binding. Epoxyazadiradione also inhibits the important hallmarks of cancer such as cell proliferation, migration and angiogenesis that is probably due to inhibition of OPN, VEGF, Cox2 and MMP-9 expression and activation. Taken together, epoxyazadiradione suppresses cell migration, angiogenesis and breast tumor growth through downregulation of PI3K/Akt-mediated mitochondrial depolarization and induction of caspase-dependent apoptosis and blocking of AP-1 activation and expression of pro-angiogenesis and metastasis genes (Fig. 7h).

Neem contains several limonoids (triterpenoids) that showed a considerable research interest in recent years. Several reports showed that it has potent anti-oxidant, anti-proliferative, anti-inflammatory and insecticide effects [19, 50]. Kikuchi et al. have isolated 35 neem limonoids including 15 azadiradione type and evaluated their cytotoxic activity against different cancer cell lines [17]. Previous data showed that neem limonoids azadirachtin and nimbolide induce mitochondria-mediated apoptosis in human cervical cancer, HeLa cells [14]. However, we found that azadirachtin is less cytotoxic in breast cancer cells suggesting that activity of these limonoids are cancer cell type specific. It has been also shown that neem oil limonoids induce p53 independent apoptosis and autophagy [51]. In our study, we have comparatively evaluated the cytotoxic activity of 10 major neem limonoids in TNBC and ER+ breast cancer cells. We found that epoxyazadiradione, a derivative of azadiradione exhibits most potent cytotoxic activity among 10 different limonoids. Epoxyazadiradione shares the same structural scaffold of azadiradione but differ in that it has an epoxide group instead of alkenyl group (Fig. 1a).

During apoptosis, disturbance of mitochondria homeostasis is linked with cancer progression [52]. While apoptosis, the expression of Bax and Bad are upregulated whereas Bcl2 expression is dowregulated which further activate mitochondria-mediated apoptotic pathway which in turn release cytochrome C, followed by caspase 9 and 3 activation leading to PARP cleavage [30, 53]. Moreover, apart from Caspase-dependent apoptosis, ROS are known to play crucial role in apoptosis. There are several anti-cancer drugs like taxol and etoposide induce apoptosis through upregulation of intracellular ROS [54–56]. In addition to this, several studies demonstrate that mitochondrial apoptosis inducing factor (AIF) which translocate to nucleus upon apoptotic signals and induce chromatin condensation and fragmentation, play an important role in programmed cell death [57]. In agreement with these results, our findings demonstrate that epoxyazadiradione induce apoptosis in both TNBC and ER+ breast cancer cells through disturbance of mitochondrial membrane potential and activation of Caspase 9 and 3-mediated PARP cleavage. However, epoxyazadiradione does not affect either of the intracellular ROS level or translocation of AIF into the nucleus.

Various signaling molecules and cytokines such as OPN, VEGF, Flk1, Cox2 and MMP-9 play an important role during process of tumor angiogenesis and metastasis [37, 58–60]. At the time of metastasis, tumor cell secretes mettallomatrix proteins (MMPs) which help in the degradation of extracellular matrix (ECM) that allows tumor cells to invade into the surrounding tissues [61]. Targeting tumor angiogenesis is an important therapeutic aspect in the regulation of tumor progression [62]. Therefore, controlling tumor angiogenesis may provide prolonged survival of cancer patients. Our results further revealed that epoxyazadiradione attenuates breast cancer cell migration and endothelial cell tube formation. Moreover, our data showed that epoxyazadiradione did not have any role on the migration potential of MDA-MB-231 cells significantly in the presence of mitomycin C, a cell cycle blocker indicating that the observed migratory effect is not due to proliferation. Further, it inhibits the expression and activation of pro-angiogenic and pro-metastatic molecules like OPN, VEGF, Flk1, Cox2 and MMP-9. Thus epoxyazdiradione effectively inhibits the various hallmarks associated with aggressive breast cancer growth.

It has been shown that PI3K/Akt pathway is generally active in most of the cancer types. Constitutive activation of PI3K/Akt pathway plays a crucial role in cell growth, survival, migration and invasion [63]. Further, this pathway protects the cancer cells against apoptosis [28, 30, 48]. Our findings demonstrate that epoxyazadiradione attenuates PI3K/Akt pathway. Further, using selective Akt inhibitor, perifosine or overexpression of Akt1 demonstrates that it regulates breast cancer cell migration, angiogenesis and induces apoptosis through PI3K/Akt pathway. Next, our results also showed that epoxyazadiradione downregulates the AP-1-DNA binding in these cells. Our in vitro findings also supported by in vivo data using NOD/SCID mice where epoxyazadiradione showed significant reduction in breast tumor growth and angiogenesis.

## Conclusion

We showed for the first time that epoxyazadiradione, a natural compound derived from neem inhibits PI3K/Akt pathway, induces apoptosis and suppresses migration, angiogenesis and breast tumor growth. These findings suggest a strong rationale for investigating the chemoprevention property of epoxyazadioradione with special emphasis to the management of breast cancer.

## Additional files

Additional file 1: Figure S1. Epoxyazadiradione inhibits breast cancer cell viability. **Figure S2.** Epoxyazadiradione induces cell death through ROS and AIF independent pathway in MCF-7 cells. **Figure S3.** Epoxyazadiradione attenuates breast cancer cell migration through downregulation of PI3K/Akt pathway. **Table S1.** List of antibodies used for western blot and immunofluorescence. **Table S2.** IC_50_ of epoxyazadiradione in breast cancer (MDA-MB-231 and MCF-7) cells. (PDF 3912 kb)

## Abbreviations

AIF: apoptosis inducing factor
ATCC: American type culture collection
EMSA: electrophoretic mobility shift assay
Epoxy: epoxyazadiradione
ER: estrogen receptor
HER2: human epidermal growth factor receptor 2
HUVECs: human umbilical vein endothelial cells
IVIS: in vivo imaging system
MMPs: matrix metalloproteinases
NAC: N-acetyl-cysteine
NOD/SCID: non-obese diabetic/severe combined immunodeficient
OPN: Osteopontin
PI3K: phosphoinositide 3-kinase
ROS: reactive oxygen species
TNBC: triple-negative breast cancer
VEGF: vascular endothelial growth factor
