# Supplementary material for: Epoxyazadiradione suppresses breast tumor growth through mitochondrial depolarization and caspase-dependent apoptosis by targeting PI3K/Akt pathway
Source: BMC Cancer. 2018 Jan 8;18:52. doi: 10.1186/s12885-017-3876-2 (PMC5759831; doi:10.1186/s12885-017-3876-2)
Supplement: Supplementary file 1 — Epoxyazadiradione inhibits breast cancer cell viability. Figure S2. Epoxyazadiradione induces cell death through ROS and AIF independent pathway in MCF-7 cells. Figure S3. Epoxyazadiradione attenuates breast cancer cell migration through downregulation of PI3K/Akt pathway. Table S1. List of antibodies used for western blot and immunofluorescence. Table S2. IC50 of epoxyazadiradione in breast cancer (MDA-MB-231 and MCF-7) cells. (PDF 3912 kb) [file 12885_2017_3876_MOESM1_ESM.pdf]

Additional File-1

Epoxyazadiradione Suppresses Breast Tumor Growth Through Mitochondrial Depolarization and Caspase-Dependent Apoptosis by Targeting PI3K/Akt Pathway

Dhiraj Kumar[1], Saikat Haldar[2#], Mahadeo Gorain[1#], Santosh Kumar[3], Fayaj A. Mulani[2], Amit S. Yadav[1], Lucio Miele[4], Hirekodathakallu V. Thulasiram[2], Gopal C. Kundu[1]*

[1]Laboratory of Tumor Biology, Angiogenesis and Nanomedicine Research, National Centre for Cell Science (NCCS), Pune 411007, India

[2]Chemical Biology Unit, Division of Organic Chemistry, CSIR-National Chemical Laboratory, Pune 411008, India

[3]Department of Biochemistry and Molecular and Cellular Biology, Georgetown University Medical Center, Washington D.C. 20057, USA

[4]Department of Genetics, LSU Health Sciences Center, New Orleans, Louisiana, 70112, USA

[#]These authors have contributed equally

Competing financial interests: The authors declare no competing financial interests

Running title: Epoxy attenuates breast tumor growth

***Correspondence**: Laboratory of Tumor Biology, Angiogenesis and Nanomedicine Research, National Centre for Cell Science (NCCS), Pune 411007, India; Email: kundu@nccs.res.in

Email ID of Authors:

Dhiraj Kumar: dhirajbiot786@gmail.com
Saikat Haldar: saikatbabu@gmail.com
Mahadeo Gorain: mahadeo.gorain@gmail.com
Santosh Kumar: kumarsant@gmail.com
Fayaj A Mulani: mulanifayaj@gmail.com
Amit S Yadav: amit03@nccs.res.in
Lucio Miele: lmiele@lsuhsc.edu
Hirekodathakallu V. Thulasiram: hv.thulasiram@ncl.res.in
Gopal C. Kundu: kundu@nccs.res.in

Supplementary Table S1: List of antibodies used for western blot and immunofluorescence

Name of Antibody	Catalog No.	Company Name	Source	Application
Bax	sc-526	Santa Cruz	Rabbit	WB, IP, IF, IHC(P) and ELISA
Bad	sc-8044	Santa Cruz	Mouse	WB, IP, IF, IHC(P) and ELISA
Bcl_2	sc-492	Santa Cruz	Rabbit	WB, IP, IF, IHC(P) and ELISA
Cox2	sc-1747	Santa Cruz	Goat	WB, IP, IF, IHC(P) and ELISA
p-Akt1/2/3	sc-7985	Santa Cruz	Rabbit	WB, IP, IF, IHC(P) and ELISA
Akt1/2	sc-1619	Santa Cruz	Goat	WB, IP, IF, IHC(P) and ELISA
Flk1	sc-6251	Santa Cruz	Mouse	WB, IP, IF and IHC(P)
VEGF	sc-1876	Santa Cruz	Goat	WB, IP, IF, IHC(P) and ELISA
c-Jun	sc-45	Santa Cruz	Rabbit	WB, IP, IF and ELISA
c-Fos	sc-52	Santa Cruz	Rabbit	WB, IP, IF, IHC(P), FCM and ELISA
AIF	sc-13116	Santa Cruz	Mouse	WB, IP, IF, IHC(P), FCM and ELISA
p-Akt	4060	Cell Signaling	Rabbit	WB, IP, IF, F and IHC
PI3K p85	4257	Cell Signaling	Rabbit	WB and IP
p-PI3K p85	4228	Cell Signaling	Rabbit	WB and IP
Cleaved Caspase-9	9508	Cell Signaling	Mouse	WB
Cleaved Caspase-3	9664	Cell Signaling	Rabbit	WB, IP, IF, F and IHC
PARP	9542	Cell Signaling	Rabbit	WB
OPN	Ab36125 (M)	Abcam	Goat	WB

Supplementary Table S2: IC_{50} of epoxyazadiradione in breast cancer (MDA-MB-231 and MCF-7) cells

Time	IC_{50} (μM) in MDA-MB-231	IC_{50} (μM) in MCF-7
24 h	95.69 $\pm$ 1.12	114.81 $\pm$ 1.14
48 h	80.71 $\pm$ 1.21	109.64 $\pm$ 10
72 h	66.93 $\pm$ 1.42	84.72 $\pm$ 1.13
96 h	66.86 $\pm$ 1.63	67.10 $\pm$ 1.05

SUPPLEMENTARY FIGURE LEGENDS:

Supplementary Figure 1: Epoxyazadiradione inhibits breast cancer cell viability. (**a**) ER+ breast cancer, MCF-7 cells were seeded into 96-well plate at density of 1 x 10^4 cells/200 µl and treated in absence or presence of ten major neem limonoids compounds (**1**: Epoxyazadiradione; **2**: Azadiradione; **3**: 17β-hydroxyazadiradione; **4**: Gedunin; **5**: Nimbin **6**: 6-Deacetylnimbin; **7**: Salannin; **8**: 3-Deacetylsalannin; **9**: Azadirachtin A; **10**: Azadirachtin B) at 100 and 200 µM concentrations for 24 h. Further, cell viability was determined using MTT assay and data were analyzed statistically and represented graphically. (**b** and **c**) MDA-MB-231 and MCF-7 cells were treated with epoxyazadiradione (0-200 µM) for 48 h, 72 h and 96 h and the cell viability was analyzed using MTT assay. (**d**) Normal human mammary epithelial cells, MCF-10A (1 x 10^4) were seeded into 96-well plate and treated with increasing concentration of epoxyazadiradione (0-200 µM) for 24 h and 48 h. Cell viability was determined using MTT assay, analyzed statistically and represented into line graph. (**e**) MCF-7 cells were treated with epozyazadiradione (0-150 µM) for 24 h, stained with propidium iodide (PI) and analyzed the cell cycle distribution by FACSCalibur cytometer. Values are represented in mean ± SEM of three independent experiments.

Supplementary Figure 2: Epoxyazadiradione induces cell death through ROS and AIF independent pathway in MCF-7 cells. (**a**) MCF-7 cells were treated with increasing concentrations of epoxyazadiradione and immunofluorescence staining was performed using phalloidin FITC or anti-AIF antibody and analyzed by confocal microscopy. Scale bar represents 10 µm. (**b**) Bar graph represents the TUNEL positive cells upon epoxyazadiradione treatment in MDA-MB-231. Data are represented in mean ± SEM of three independent experiments. (**c**) MCF-7 cells were treated with epoxyazadiradione in dose-dependent manner, stained with DHE at 37ºC for 20 min and analyzed by flow cytometry. The percentage of DHE staining was quantified and represented graphically. (**d**) MDA-MB-

231 cells were pretreated with caspase inhibitor (40 μM) for 1 h, wounded with constant width and treated with either epoxyazadiradione (20 μM) or mitomycin C (10 μg/ml) or in combination of both for further 12 h. Photographs of wound were taken at T = 0 and 12 h. Migrated distance were measured using Image-Pro plus software and analyzed statistically and represented graphically using Sigma Plot software (*, $p < 0.015$; ***, $p < 0.0001$ by one-way ANOVA with untreated control cells).

Supplementary Figure 3: Epoxyazadiradione attenuates breast cancer cell migration through downregulation of PI3K/Akt pathway. (**a**) MCF-7 cells were treated with epoxyazadiradione in dose-dependent manner for 24 h and the expressions of p-Akt and c-Jun were analyzed by immunoblot. (**b**) MDA-MB-231 cells were transiently transfected with pcDNA6-HA-Akt1 for 48 h and the expression level of p-Akt1, Akt1 or HA were analyzed by western blot. Actin was served as loading control. (**c**) MDA-MB-231 cells were pretreated with or without epoxyazadiradione (20 μM) or perifosine (10 μM). In separate experiments, these cells were transiently transfected with Akt1 as described above followed by epoxyazadiradione (20 μM) treatment for 12 h. These cells were added on upper portion of Transwell chamber and incubated for another 12 h at 37ºC. Migrated cells to the lower side of Transwell membrane were stained with 5% Crystal Violet and photographed (10X magnification).

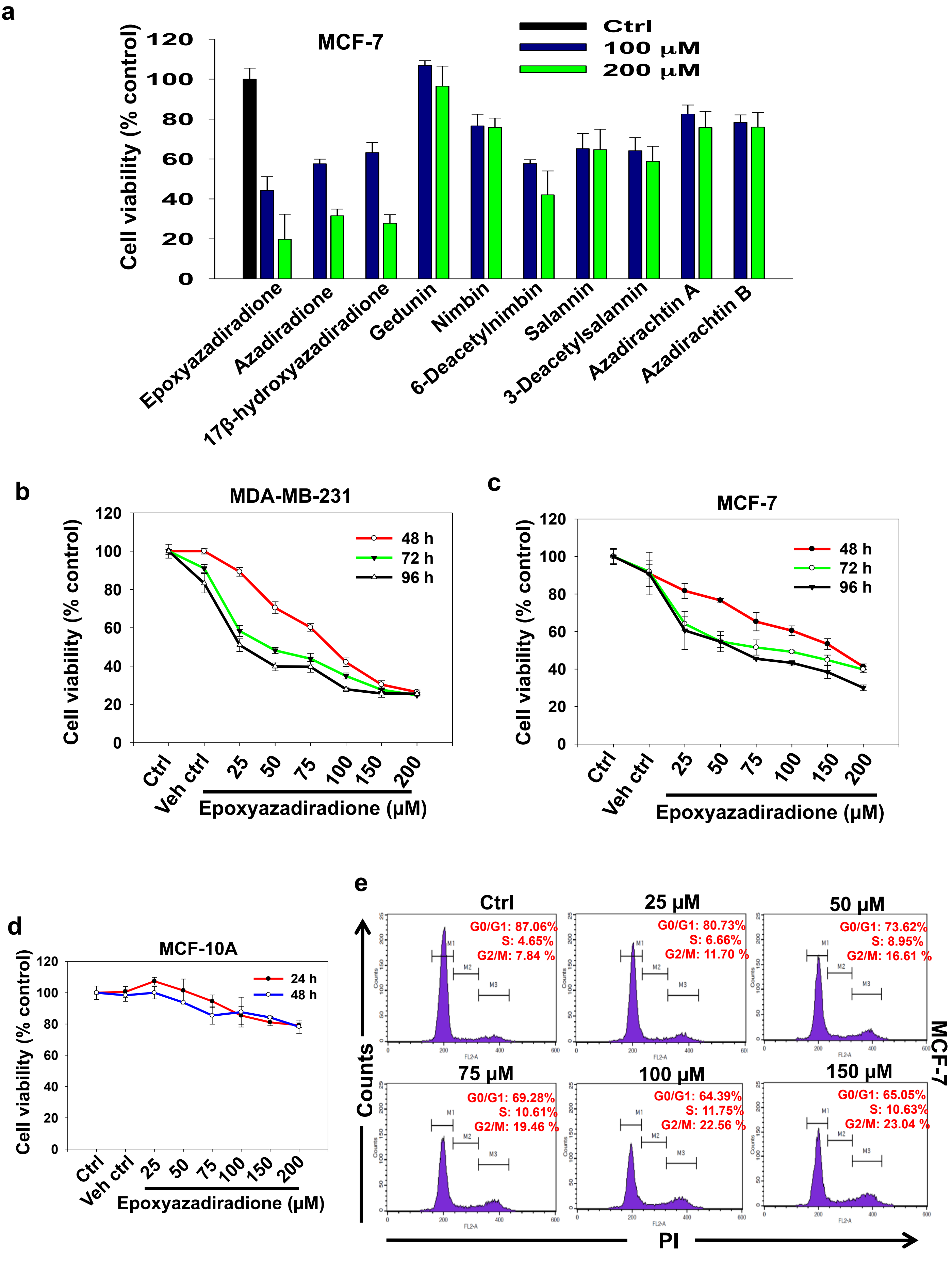

Supplementary Figure: 1 Kumar et al

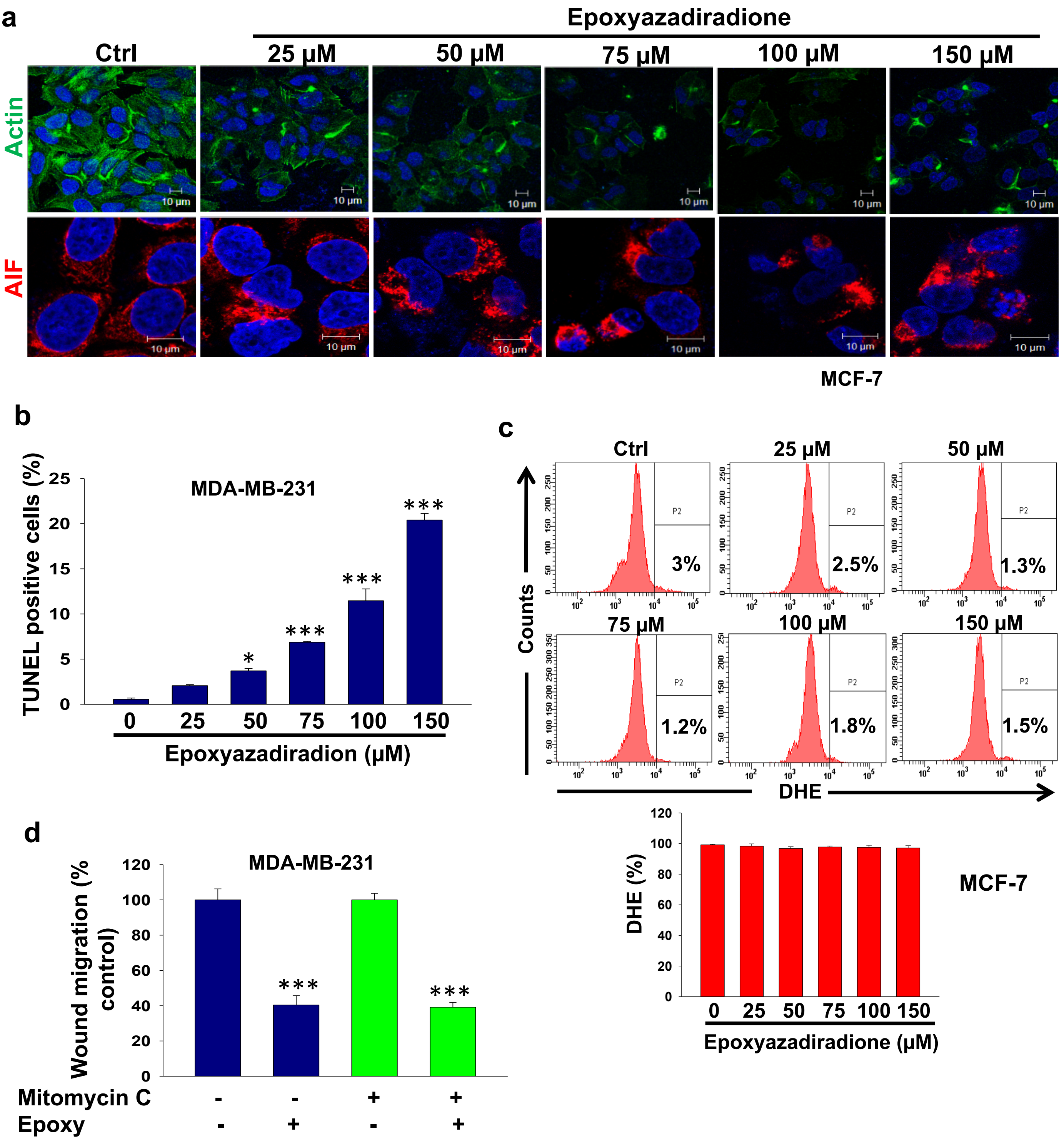

Supplementary Figure: 2 Kumar et al

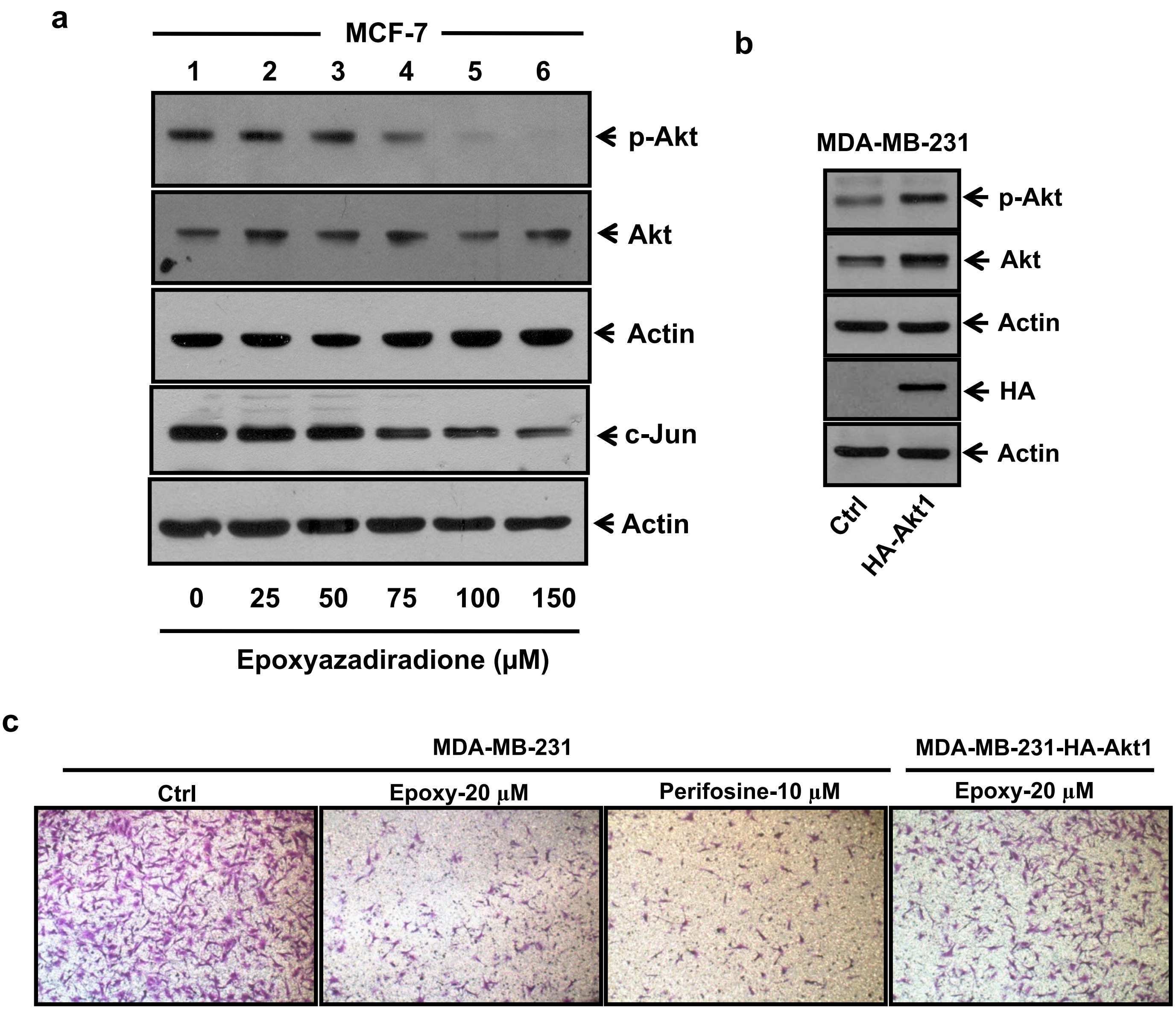

Supplementary Figure: 3 Kumar et al
